# “Looking at” Negation: Faster Processing for Symbolic Rather Than Iconic Representations

**DOI:** 10.1007/s10936-021-09797-w

**Published:** 2021-09-03

**Authors:** Isabel Orenes

**Affiliations:** Departamento de Psicología Básica I, Universidad Nacional de Educación a Distancia (UNED), C/ Juan del Rosal, 10, 28040 Madrid, Spain

**Keywords:** Negation, Processing, Representation, Compound sentences, Eye-tracking

## Abstract

Many studies have shown the double processing of negation, suggesting that negation integration into sentence meaning is delayed. This contrasts with some researches that have found that such integration is rather immediate. The present study contributes to this debate. Affirmative and negative compound sentences (e.g., “because he was *not* hungry, he did *not* order a salad”) were presented orally in a visual world paradigm while four printed words were on the screen: salad, no salad, soup, and no soup. The eye-tracking data showed two different fixation patterns for negative causal assertions, which are linked to differences in the representation and inferential demands. One indicates that negation is integrated immediately, as people look at the explicit negation (e.g., no salad) very early. The other, in which people look at the alternate (e.g., soup) much later, indicates that what is delayed in time is the representation of the alternate. These results support theories that combine iconic and symbolic representations, such as the model theory.

## Introduction

Negation is present in all natural and artificial languages, and children from an early age use and understand it. Yet, studies show that negative sentences are more difficult to process than affirmative sentences (Carpenter & Just, 1975; Clark & Chase, 1972; Farshchi et al., 2019; Greco, 2020; Kaup & Dudschig, 2020; Trabasso et al., 1971; Wason & Johnson-Laird, 1972; Wason & Jones, 1963). This asymmetry between both types of sentence is often known as the negation effect and has interested psychologists, linguists and philosophers alike.

Probably, the most accepted proposal in literature to explain the negation effect is its double processing. This idea stems from the logician Bertrand Russell who wrote that 'when I say, "this is not blue" I somehow consider it to be blue first and then reject it, considering it a colour other than blue' (Russell, 1948). This hypothesis has been taken up by different theories. According to the embodied cognition account, negation cannot be explicitly represented given that it is a linguistic operator that has no direct experiential simulation. As a result, the processing of a negative sentence (e.g., “the door is not open”) involves two steps during comprehension (Kaup & Zwaan, 2003): first, the representation of the negated situation (e.g., an open door) and then the actual situation (e.g., a closed door). Negation meaning would be implicitly captured in the deviations between these two simulations. Thus, according to this view, negation is difficult because, unlike affirmative sentences (e.g., “the door is closed”), which directly and exclusively represent the actual situation (e.g., a closed door), it calls for two mental simulations (Dudschig & Kaup, 2020a; Kaup et al., 2006). Recently, this two-step theory has been supplemented by mechanistic proposals that suggest that negation operates through processes associated with cognitive control functions; in particular, with conflict monitoring and inhibition (Dudschig & Kaup, 2018, 2020b; see also Beltrán et al., 2019, 2021; Liu et al., 2020a, 2020b). Within this new framework, it has been suggested that, in some circumstances, comprehenders can up-regulate control processes to jump directly to the meaning of negation (Dudschig & Kaup, 2018). Therefore, even though two simulation steps might be the default way to process negative sentences according to this theory, they are not mandatory.

The theory of mental models (the “model theory” for short; see Johnson-Laird, 2006) is consistent with the double processing of negation and claims that our representations are as iconic as possible. In other words, the structure of our mental representations (mental models) is analogue to the structure of the world under description (see Peirce 1931–1958). This theory is in line with the sensorimotor representations that the embodied cognition account endorses (Barsalou, 2012; Glenberg et al., 1999), but it also allows for the use of combinations with symbolic representation, and this is the case for negation (for a review, see Khemlani et al., 2012). Accordingly, it postulates that negative sentences, e.g., “the door is not blue” could be represented by a simulation of the negated situation (e.g., a blue door) plus a symbol that represents negation. Moreover, it predicts different representations for negation depending on the type of negated predicate, whether binary (e.g., open) or multiple (e.g., red). Both cases start with the representation of the negated situation, but they differ in the representation format of the actual situation. For the binary predicate (e.g., “the door is not open”), negation is expected to bring forth a representation of its alternate (e.g., an iconic simulation of a closed door), while for the multiple predicate (e.g., “the door is not blue”), which has many alternates (e.g., red, green, yellow, etc.), an iconic plus symbolic representation is expected. The reason for the combined representation is that the more alternates for a negated predicate, the greater the overload on the working memory to represent them (e.g., Orenes et al., 2014; see also Beltrán et al., 2008; Espino & Byrne, 2018; Khemlani et al., 2014). Nonetheless, both types of representations can be modulated by pragmatics. Multiple predicates can be converted into binary by highlighting one of the possibilities or the negation of binary predicates can block the representation of the alternate, e.g., ‘not open exactly the correct set of boxes’ in which people should retain “open” together with the negation marker rather than representing the alternate since the boxes could be already closed. The model theory also predicts that the context could accelerate the processing of negation, specifically, when individuals have already constructed the negated situation by a previous context, and they should interpret negation straightforwardly. Indeed, the main use of negation is to deny misconceptions (Wason, 1965). This would lead to process negation just as fast as the corresponding affirmation.

The double processing of negation is in general rejected by pragmatically-oriented researchers, which focus on the fact that negation is integrated immediately, and not at a delayed stage of processing (Glenberg et al., 1999; Nieuwland & Kuperberg, 2008; Wason, 1965; Xiang et al., 2020). From this point of view, it has been claimed that the actual situation is directly represented and that the negated situation is not necessary to comprehend negation (Anderson et al., 2010; Giora, 2006; Giora et al., 2007; Giora et al., 2009; Huette & Anderson, 2012; Tian & Breheny, 2018; Tian et al., 2010). Negation is highly context-dependent and when it is presented in an adequate context, negation can be even faster than affirmative sentences (see Johnson-Laird & Tridgell, 1972; Khemlani et al., 2012; Orenes et al., 2021). Thus, artificial contexts used in research could explain the negation effect. Nevertheless, there are some studies that have found that negative sentences take longer to be processed than affirmative ones even when they are presented in a supportive context (see Darley et al., 2020; Orenes et al., 2015).

One of the evidences for the double processing of negation has been that, in some paradigms, its integration into the comprehension process is delayed between 750 and 1500 ms (Hasson & Glucksberg, 2006; Kaup et al., 2006). Orenes et al (2014) explored the impact of context on the time course of negation processing in a visual-world eye-tracker study. The idea behind this paradigm is that people explore the visual field guided by the meaning of what they are processing in real time. In other words, a correspondence is assumed between what is more active in working memory and where the visual attention is focused (Cooper, 1974; Just & Carpenter, 1976; for reviews see Huettig et al., 2011; Tanenhaus et al., 1995). Therefore, eye-tracking is a suitable tool to study what and when people represent while comprehending a sentence. In Orenes et al. (2014), participants heard negative sentences (e.g., “the figure is not blue”) that followed binary contexts (e.g., “the figure could be blue or yellow”), while their looks towards four colour figures (blue, yellow, red and green) on the screen were registered. Results showed that they looked at the figure corresponding to the actual situation through the alternate (e.g., a yellow figure) in a relatively late time window, around 1500 ms*,* confirming thereby that negation integration is delayed. Critically, before the fixation on the alternate, no increase of the looks on the negated situation (e.g., a blue figure) was observed, which suggested that a full-fledged representation of the negated situation might not be necessary to understand negation. An alternative hypothesis could be that the participants have already constructed the negated situation previously since all colour figures were presented on the screen from the beginning or even that the representation of the negated situation is very fast, and not reflected in eye movements.

However, negative sentences are not always this informative about the alternate. In the same study, when individuals heard negative sentences after multiple contexts (e.g., “the figure could be blue or yellow or red or green”), they looked at the figure corresponding to the negated situation (e.g., a blue figure) from 400 to 500 ms onwards. This result suggests an initial simulation step devoted to representing the negated situation as if negation had been actually removed from the sentence, thus being consistent with the two-step simulation theory. Yet, in line with the mental model theory, it could be equally interpreted as reflecting a mixed representation, composed of both a symbolic marker of negation and the simulation of the negated situation. To decide between these alternative interpretations, Orenes et al. (2021) asked participants to look at printed words, instead of pictures. After hearing negative sentences, they found that participants looked since very early on at the negative phrase (e.g., no blue), neglecting the affirmative word (e.g., blue). Apparently, individuals preferred to fix on the representation that explicitly included the negation, supporting then the model theory. More importantly, this preference for an explicit negation suggests an immediate integration of the negative operator (see Nieuwland & Kuperberg, 2008; Tian et al., 2010). However, the question that arises is whether this fast processing of negation reflects either its meaning integration or uniquely a superficial lexical matching.

The main hypothesis of the present paper is that negation is indeed integrated quickly and that what takes an extra processing time is the representation of its alternate, as it is time-consuming to replace negation (e.g., “the figure is not blue”) by an equivalent affirmative representation (e.g., “the figure is yellow”; see Johnson-Laird & Tridgell, 1972). To test this hypothesis, the visual world paradigm was used. As in Orenes et al. (2021), in the present experiment, printed words were used instead of pictures. Previous literature has shown similar results for both formats in the visual world paradigm (McQueen & Viebahn, 2007; Primativo et al., 2016). The printed word format may be more sensitive to investigate phonological and orthographic processing than the picture format and less sensitive to semantic processing (Huettig & McQueen, 2011; Salverda & Tahenhaus, 2010). Nevertheless, visual information such as pictures can impede negation processing (e.g., Orenes & Santamaría, 2014; see also Knauff & Johnson-Laird, 2002). Moreover, prior research has shown that, when hearing a negative sentence (e.g., “the figure is not blue”), participants prefer to look at the negative phrase (e.g., no blue) than an image of a blue figure with a cross through it (Orenes et al., 2019). The printed words, particularly the target that is attended by subjects, would be an external support for the processing of working memory in a similar way as target pictures support it when they are presented on the screen. In the present study, compound sentences that are not frequently studied were presented orally: affirmative and negative causal assertions, e.g., “because he was (not) hungry, he did (not) order a salad” and counterfactuals e.g., “if he had (not) been hungry, he would (not) have ordered a salad,” while four printed words or phrases were shown on the screen: salad, no salad, soup, no soup and eye movements were registered. In the visual word paradigm, oral language, the visual context (in this study, the printed words) and the eyes interact. In the present experiment, there is a high correspondence between what people hear and what they see. Then, it is expected that eye movements are guided mainly by the meaning of the sentence that is being heard (see Altmann & Kamide, 1999; Duñabeitia et al., 2009; Huettig & Altmann, 2011) rather than by other linguistic properties, such as, phonological or orthographic features, that are not manipulated here (see Ito, 2019).

For the present study, the following predictions were made. First, participants will look at the affirmative mentioned word (e.g., salad) for affirmative causal assertions (e.g., “because he was hungry, he ordered a salad”). Secondly, for negative causal assertions (e.g., “because he was not hungry, he did not order a salad”), it is expected that people will show two ways of negation processing, by fixating either on the alternate (e.g., soup) or on the explicit negation (e.g., no salad). The latter is expected because the sentences that are used in the present experiment are not binary, that is, its negation, e.g., “he did not order a salad” has no available alternate, but many possible ones. Nonetheless, the task or the visual context (salad, no salad, soup, no soup) could make an alternate of the negation more available and thus people represented it (e.g., soup). It is also predicted that the looks towards the explicit negation (e.g., no salad) will be faster than for the alternate (e.g., soup; see Orenes et al., 2014; Johnson-Laird & Tridgell, 1972).

An alternative hypothesis is that this quick representation of negation through the explicit negation (e.g., no salad) is not an evidence of meaning integration, but it simply reflects a preference for looking at what is heard regardless of its conceptual meaning. To rule this out, affirmative and negative counterfactuals are presented. On the one hand, when people understand a negative counterfactual, such as, “If he had *not* been hungry, he would *not* have ordered a salad,” they follow a double processing similar to negation (see de Vega et al., 2007; Hasson & Glucksberg, 2006). They envisage two possibilities: the conjecture (the counterfactual situation that corresponds to the negated situation for negation), e.g., “he was *not* hungry, and he did *not* order a salad” and its opposite, the presupposed facts (the factual situation that corresponds to the actual situation for negation), e.g., “he was hungry, and he ordered a salad” (e.g., Thompson & Byrne, 2002). In this case, if people look at what they hear based on superficial processes, they would look at just the explicit negation (e.g., no salad) immediately, the same as with any negative sentence, however it is predicted that they should look at the factual situation (e.g., salad) more likely than the conjecture (see Orenes et al., 2021). This could indicate that the looks are guided by the conceptual meaning. On the other hand, when people understand affirmative counterfactuals, such as, “If he had been hungry, he would have ordered a salad,” they represent the conjecture, e.g., “he was hungry, and he ordered a salad” and the factual situation or the presupposed facts, e.g., “he was *not* hungry, and he did *not* order a salad” (Byrne, 2005; 2016). As seen, affirmative counterfactuals do not have the explicit negation that negative counterfactuals have, but the factual situation refers to a fact that does not happen, therefore the last prediction is that people should look at the explicit negation (e.g., no salad), although it was not heard. This tests whether the representation of the explicit negation is a matter of semantics.

The causal assertions and counterfactuals used in this study have a similar grammatical structure, with an antecedent and a consequence, but the first is expressed in an indicative mood and the second in a subjunctive mood. For causal assertions, there is a match between what is mentioned and its meaning, and consequently there is no easy way to identify whether eye gaze corresponds to a shallow or full comprehension. In contrast, for counterfactuals, what is mentioned corresponds to the imagined situation, while the factual situation is not explicit through discourse. This difference between what is asserted and presupposed allows studying whether the eye gaze is guided by the sentence meaning or by superficial features. It is important to note that various levels of representation are involved in the comprehension processes. First, people build a text-based representation through the meaning of words and the grammatical relations amongst them, and second they construct a mental model of the described situation (see Johnson-Laird, 1983; Zwaan & Radvansky, 1998). While they just represent the facts that are described for causal assertions, they represent the conjecture beside the factual situation for the counterfactuals. Of course, the context or knowledge may modulate what is referred to in the assertion. Individual differences could arise depending on how people use the contextual clues. Eye fixations are driven by the interplay between the lexical meaning, the sentence construction and context. In sum, two main hypotheses are tested in this study: (1) negation is integrated immediately and what takes an extra processing time is the representation of its alternate. To test this hypothesis, causal assertions were presented; (2) It is necessary to discard that the fast integration of negation is not due to superficial features, but its meaning. To this end, counterfactuals were used.

## Method

### Participants

The participants were 30 university students (24 women). Their average age was 18 years, ranging between 17 and 25 years. The participants were native Spanish speakers and they all reported normal or corrected to normal vision (glasses or contact lenses). They participated in the experiment in exchange for course credits.

### Materials and Design

Participants received 36 vignettes (adapted from Orenes et al., 2021), nine trials in each condition and the order of the trials was randomized. The design was a 2 (polarity: affirmative or negative) × 2 (type of assertions: causal assertions or counterfactuals) within participants. Each vignette started with an opening scene, e.g., “Carlos finished work and went to a bar” (translated from Spanish *“Carlos acabó de trabajar y se fue a un bar”*). The second sentence contained either an affirmative causal assertion, e.g., “Because he was hungry, he ordered a salad” (*“Como tenia hambre, pidió una ensalada”*) or a negative one, “Because he was not hungry, he did not order a salad” (*“Como no tenia hambre, no pidió una ensalada”*); or an affirmative counterfactual, e.g., “If he had been hungry, he would have ordered a salad” (*“Si hubiera tenido hambre, habría pedido una ensalada”*), or a negative one, e.g., “If he had not been hungry, he would not have ordered a salad” (*“Si no hubiera tenido hambre, no habría pedido una ensalada”*). These two sentences were prerecorded and presented via a computer speaker. When the second sentence was presented, four printed words or phrases were shown on a computer screen, e.g., the affirmative mentioned word “salad” (*“ensalada”*), the negative phrase “no salad” (*“no ensalada”*), the affirmative alternate “soup” (*“sopa”*)*,* and the negative alternate “no soup” (*“no sopa”*). The third sentence concluded the trial. This last sentence was consistent with previous information, after the affirmative causal assertion or the negative counterfactual, e.g., “Carlos ordered a salad” (*“Carlos pidió una ensalada”*) and after the negative causal assertion or the affirmative counterfactual, e.g., “Carlos ordered a soup” (*“Carlos pidió una sopa”*). This third sentence replaced the four words on the screen for participants to read and press the button on a gamepad as soon as they finished reading the sentence*. *Four versions of each vignette were constructed and varied in each condition. Each participant received only one of the four possible versions (affirmative or negative causal assertions, or affirmative or negative counterfactuals).

### Procedure

The eye movements were recorded at a rate of 500 Hz, using an SR Research EyeLink II head-mounted eye-tracker, connected to a 21 color CRT for visual stimulus presentation. Procedures were implemented in SR Research Experiment Builder. Calibration and validation procedures were carried out at the beginning of the experiment and were repeated several times per session. Participants were instructed to listen to the sentences carefully and that they should not take their eyes off the screen throughout the experiment. Trials started with the presentation of a central fixation dot for drift correction while participants listened to the opening-scene sentence. Next, a display with four printed words appeared for 3 s. The printed words remained later on screen while the target sentence was heard. When the participants read the last sentence, their task was to read and press the button as quickly as possible. There was a practice block of four trials before the experiment started. Participants were tested individually in a quiet testing room and each experimental session lasted approximately 30 min.

## Results and Discussion

### Eye-Tracking Data Coding

The analysis of fixations was time-locked to the onset of the last word of the sentence, e.g., the onset of “salad,” to 3000 ms after that word. The periods were divided into 50 ms time slots and for each time slot, the number of fixations on each rectangle quadrant corresponding with each word was counted and converted into fixation probabilities. The number of fixations on each word was divided by the sum of the fixation on the four words. To avoid problems inherent to proportional data, participant averages were arcsin-transformed prior to t-test comparisons. Given that 180–200 ms are usually assumed to account for saccade programming (Martin et al., 1993), the mean of the first time-region (0–100 ms) was considered to be the baseline and was used to conduct statistical comparisons against means on each time point until 3000 ms later (for a similar method, see Huettig & Altmann, 2011; Orenes et al., 2014). A false discovery rate (FDR) thresholding procedure was used to effectively control for Type 1 error due to multiple comparisons (60 for each condition; see Genovese et al., 2002).

### T-Tests Against the Baseline of 0–100 ms

**Fig. 1:**
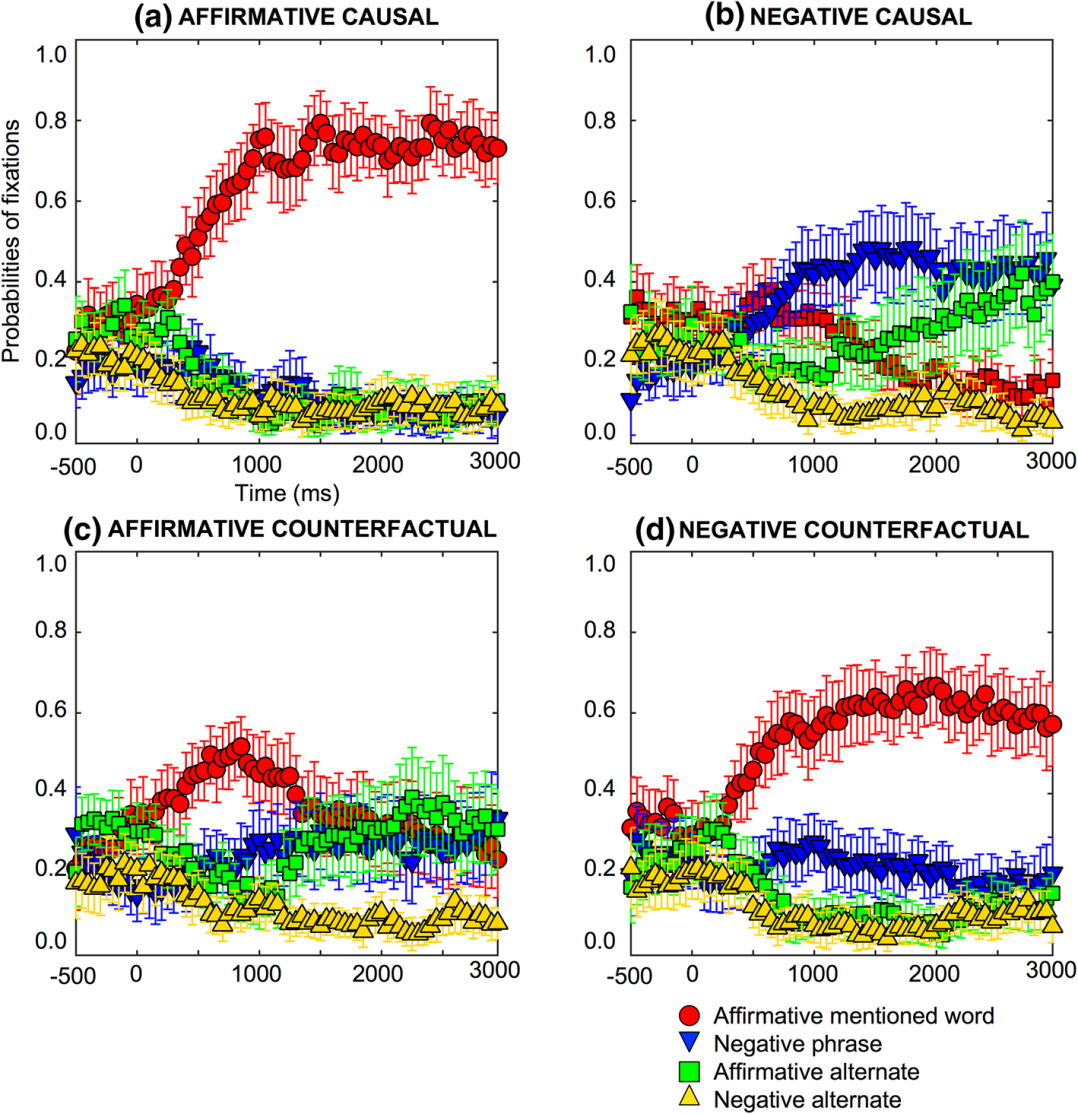
Probabilities of fixations on the affirmative mentioned word (e.g., salad), negative phrase (e.g., no salad), affirmative alternate word (e.g., soup) and negative alternate (e.g., no soup), for (a) affirmative causal assertions, (b) negative causal assertions, (c) affirmative counterfactuals, and (d) negative counterfactuals, time-locked to the onset of the first object word, e.g., “salad”. Error bars are 95% confidence intervals within participants (see Morey, 2008; O’Brien & Cousineau, 2014)

*Affirmative causal assertions*: e.g., “Because he was hungry, he ordered a salad.” Participants focused on the four words or phrases on the screen (e.g., salad, no salad, soup, and no soup) at the outset with probabilities of fixation of 0.1 to 0.4 as Fig. 1a shows. The probabilities of fixation on the affirmative mentioned word (e.g., salad) started to increase very early from 450 ms (*pFDRcorr* = 0.0086). Fixations decreased on the negative phrase (e.g., no salad, from 1500 ms*, pFDRcorr* = 0.0294), the affirmative alternate (e.g., soup, from 600 ms, *pFDRcorr* = 0.0059), and the negative alternate (e.g., no soup, from 250 ms, *pFDRcorr* = 0.0378). Hence, participants looked at the affirmative mentioned word (e.g., salad) very early in the affirmative causal assertion.

*Negative causal assertions*: e.g., “Because he was *not* hungry, he did *not* order a salad.” Participants focused on the four words on the screen with probabilities of fixation of 0.1 to 0.4, as Fig. 1b shows. The probabilities of fixation on the affirmative mentioned word (e.g., salad) started to decrease from 1800 ms (*pFDRcorr* = 0.0233). Fixations on the negative phrase (e.g., no salad) increased from 600 ms (*pFDRcorr* = 0.0394), while the affirmative alternate (e.g., soup) remained relatively constant, and showed no differences compared to the baseline throughout. Fixations decreased on the negative alternate, (e.g., no soup, from 950 ms, pFDRcorr = 0.0290). Hence, participants looked at the negative phrase (e.g., no salad) early in the negative causal assertion while they maintained their gaze on the affirmative alternate (e.g., soup) for the whole period of time.

**Fig. 2:**
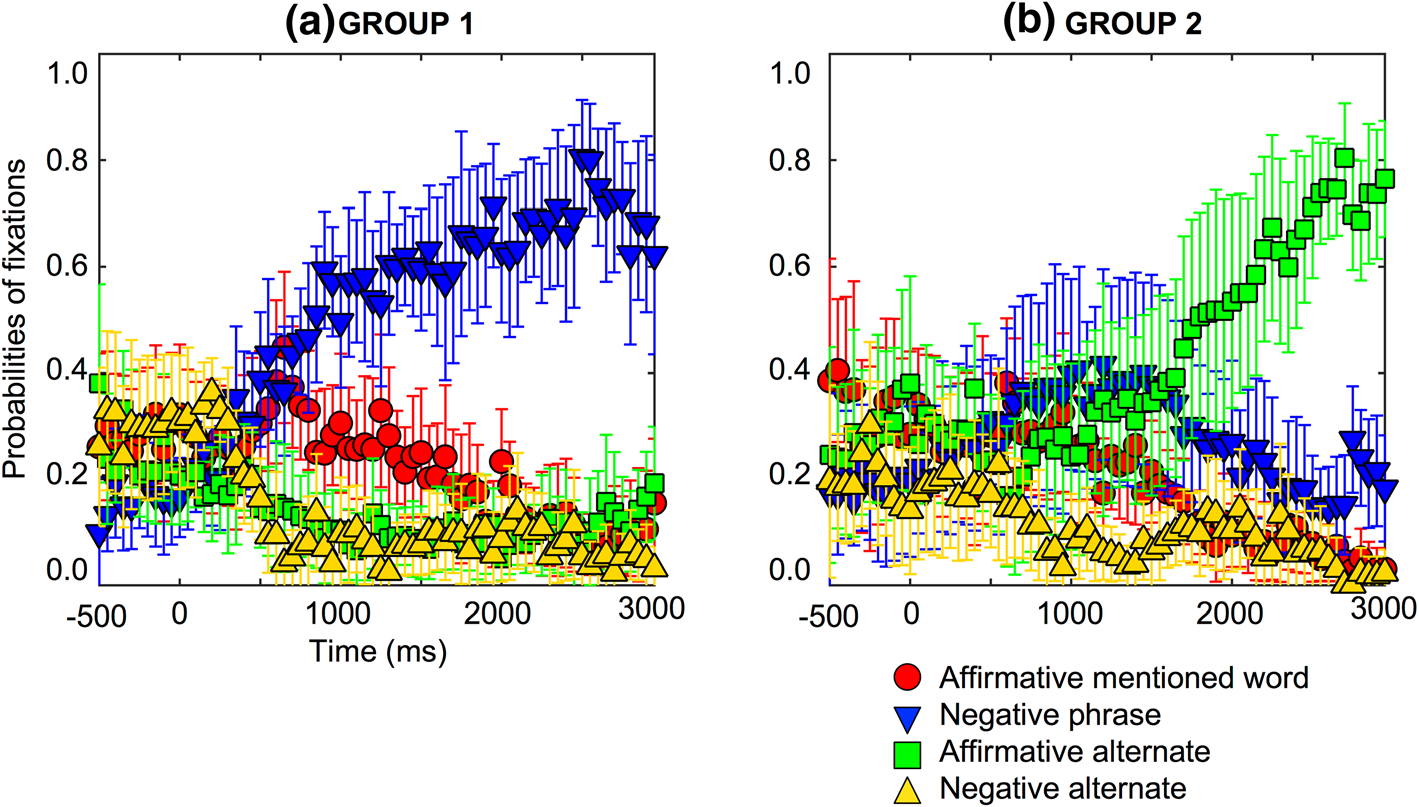
Individual difference probabilities of fixations for negative causal assertions for one subset group of 11 participants who looked at the negative phrase, e.g., “no salad” (a), and a second subset group of 11 participants who looked at the affirmative alternate, e.g., “soup” (b). Error bars are 95% confidence intervals within participants (see Morey, 2008; O’Brien & Cousineau, 2014)

Previous studies have found two ways to understand negation by the representation of the negative phrase or the affirmative alternate (see Orenes et al., 2014; Orenes et al., 2021). This was studied as per each participant and two different types of negation processing were found. The participants were split in two groups, those who looked at the negative phrase in the majority of cases (n = 11); and those who looked at the affirmative alternate in the majority of cases (n = 11). Eight participants were excluded from this analysis because they did not satisfy any of the criteria (see Fig. 2).

*GROUP 1: Pattern of fixations on the negative phrase when understanding the negative causal assertions.* Participants focused on the four words on the screen with probabilities of fixation of 0.1 to 0.4, as Fig. 2a shows. The probabilities of fixation on the affirmative mentioned word (e.g., salad) started to decrease from 2200 ms (*pFDRcorr* = 0.0156). Fixations on the negative phrase (e.g., no salad) increased from 400 ms (*pFDRcorr* = 0.0232), while the affirmative alternate (e.g., soup) remained relatively constant, and showed no differences compared to the baseline throughout. Fixations decreased on the negative alternate (e.g., no soup, from 650 ms, pFDRcorr = 0.0397). Hence, this group of participants looked at the negative phrase (e.g., no salad) very early in the negative causal assertion.

*GROUP 2: Pattern of fixations on the affirmative alternate when understanding the negative causal assertions.* Participants focused on the four words on the screen with probabilities of fixation of 0.1 to 0.4, as Fig. 2b shows. The probabilities of fixation increased on the affirmative alternate (e.g., soup, from 2300 ms, pFDRcorr = 0.0150), while the affirmative mentioned word (e.g., salad), the negative phrase (e.g., no salad), and the negative alternate, (e.g., no soup) remained relatively constant, and showed no differences compared to the baseline throughout. Hence, this group of participants looked at the affirmative alternate word (e.g., soup) very late in the negative causal assertion.

In sum, these results corroborated the first hypothesis presented in this study. On the one hand, it seems that negation is integrated very quickly, and some people look at the negative phrase very early on, while other people look at the affirmative alternate later. It seems that people need to figure out that “no salad” means “soup” in the context of the present study and this inference is time consuming (see Johnson-Laird & Tridgell, 1972). Of course, in the negative causal assertions, e.g., “Because he was *not* hungry, he did *not* order a salad” it remains unclear whether something was ordered at all and, if so, what it was, thus some people remain on the explicit negation (e.g., no salad), but not all of them showed this strategy. Others predicted what was coming and represented the affirmative alternate (e.g., soup) given that the last sentence was an affirmative sentence coherent with the target, e.g., “Carlos ordered a soup” after the negative causal assertion “Because he was *not* hungry, he did *not* order a salad.” Therefore, the own story could elicit the representation of the alternate (e.g., soup). On the other hand, both fixation patterns of negation processing were at odds with the double processing because there was no increase of looks toward the affirmative mentioned word (e.g., salad) in any of the processes of negation.

One alternative hypothesis is that the early visual attention toward the negative phrase (e.g., no salad) of negation is a matter of a shallow semantic processing (e.g., Ferreira et al., 2002; see also Barton & Sanford, 1993; Erickson & Mattson, 1981) or a heuristic processing of matching (e.g., Evans et al., 1999). In other words, people look at what they have heard addressing by superficial lexical features and without taking into account the meaning of the sentence. If this were true, they would look at the negative phrase (e.g., no salad) for negative counterfactuals the same as for the negative casual assertions. However, this hypothesis is discarded in the following lines.

*Negative counterfactuals*: e.g., “If he had *not* been hungry, he would *not* have ordered a salad.” Participants focused on the four words on the screen at the outset with probabilities of fixation of 0.1 to 0.4, as Fig. 1d shows. The probabilities of fixation on the affirmative mentioned word (e.g., salad) started to increase early on in the process, 400 ms after the mentioned word onset (*pFDRcorr* = 0.0255) and remained elevated compared to the baseline thereafter. Fixations on the negative phrase (e.g., no salad) remained relatively constant, and showed no differences compared to the baseline throughout. Fixations decreased on the affirmative alternate word (e.g., soup, from 750 ms, *pFDRcorr* = 0.0367) and the negative alternate (e.g., no soup, from 750 ms, *pFDRcorr* = 0.0323). Hence, participants looked at the affirmative mentioned word (the factual situation, e.g., salad) very early on while they maintain their stare on the negative phrase (the conjecture, e.g., no salad) the whole period of time in the negative counterfactuals. As can be observed, people did not increase their fixations towards the negative phrase although it was mentioned. Therefore, the representation of the negative phrase (the explicit negation) in the negative causal assertions is not a matching automatic response. Moreover, the participants did not replace the negative phrase by its affirmative alternate (e.g., soup), and people usually do it when the alternate is available, but it did not happen for the conjecture. Future researches should study whether this inference only happens when it refers to the factual situation rather than the conjecture, i.e., it could be related to the epistemic status given that this inference (from the explicit negation “no salad” to the alternate “soup”) takes an extra time.

It is also interesting to note that the processing of negative counterfactuals is surprisingly fast. It can be considered a sort of double negation, in so far as it mentions a negative conjecture (e.g., “he was not hungry and did not order a salad”) but communicates implicitly the opposite of what it mentions, the affirmative presupposed facts (e.g., “he was hungry and ordered a salad”). This could indicate that the slowdown time associated to the processing of negation, in general, is not related to the syntactic operator “no,” but instead could be related to its negative meaning (e.g., “no salad” for negative indicative sentences instead of “salad” for negative counterfactuals; Miller, 1962). An alternative hypothesis could be that people follow a strategy in order to simplify the comprehension and be more efficient, but it would be odd that they only used a strategy for negative counterfactuals, not for affirmatives. Wason´s (1965) account of the pragmatics of negation predicted that not-A brings A to mind, but no-one could suppose that A brings not-A to mind. The consequence of this is that the representation of the factual situation for negative counterfactual could be represented faster than for an affirmative one.

Another important difference between both types of negation is that the affirmative mentioned word (e.g., salad) is inhibited in the negative causal assertions, that is, people decreased their fixations on it, while the visual attention increased on the mentioned word for negative counterfactuals. This means that the inhibition of the affirmative mentioned word is related to the denial of negation, but not to the negation operator itself. This is important as there is a new approach to negation as a general cognitive mechanism of inhibition (Beltrán et al., 2019; de Vega, et al. 2016; García-Marco et al., 2019). All these exceptions make negative counterfactual a special negation. In order to paint the whole picture, the affirmative counterfactuals were analyzed.

*Affirmative counterfactuals*: e.g., “If he had been hungry, he would have ordered a salad.” Participants focused at the outset on the four words on the screen with probabilities of fixation of 0.1 to 0.4, as Fig. 1c shows. The probabilities of fixation on the affirmative mentioned word (e.g., salad) increased from 550 ms (*pFDRcorr* = 0.05), and next decreased with no significant differences from the baseline from 1100 ms onwards (*pFDRcorr* = 0.0914). Fixations on the negative phrase (e.g., no salad) remained relatively constant, and showed no differences compared to the baseline throughout. Fixations decreased on the affirmative alternate word (e.g., soup, from 600 ms, *pFDRcorr* = 0.0060), and then increased with no significant differences from the baseline from 1300 ms (*pFDRcorr* = 0.0830). This fixation pattern is opposite to the affirmative mentioned word that increased first and then decreased. Fixations decreased on the negative alternate (e.g., no soup, from 650 ms, *pFDRcorr* = 0.0409). Hence, participants looked at the conjecture (e.g., salad) and the factual situation by the negative phrase (e.g., no salad) and the affirmative alternate (e.g., soup) in the affirmative counterfactual. This finding shows that fixations are able to detect the double processing of counterfactuals, however it is not corroborated for negative causal assertions.

**Fig. 3:**
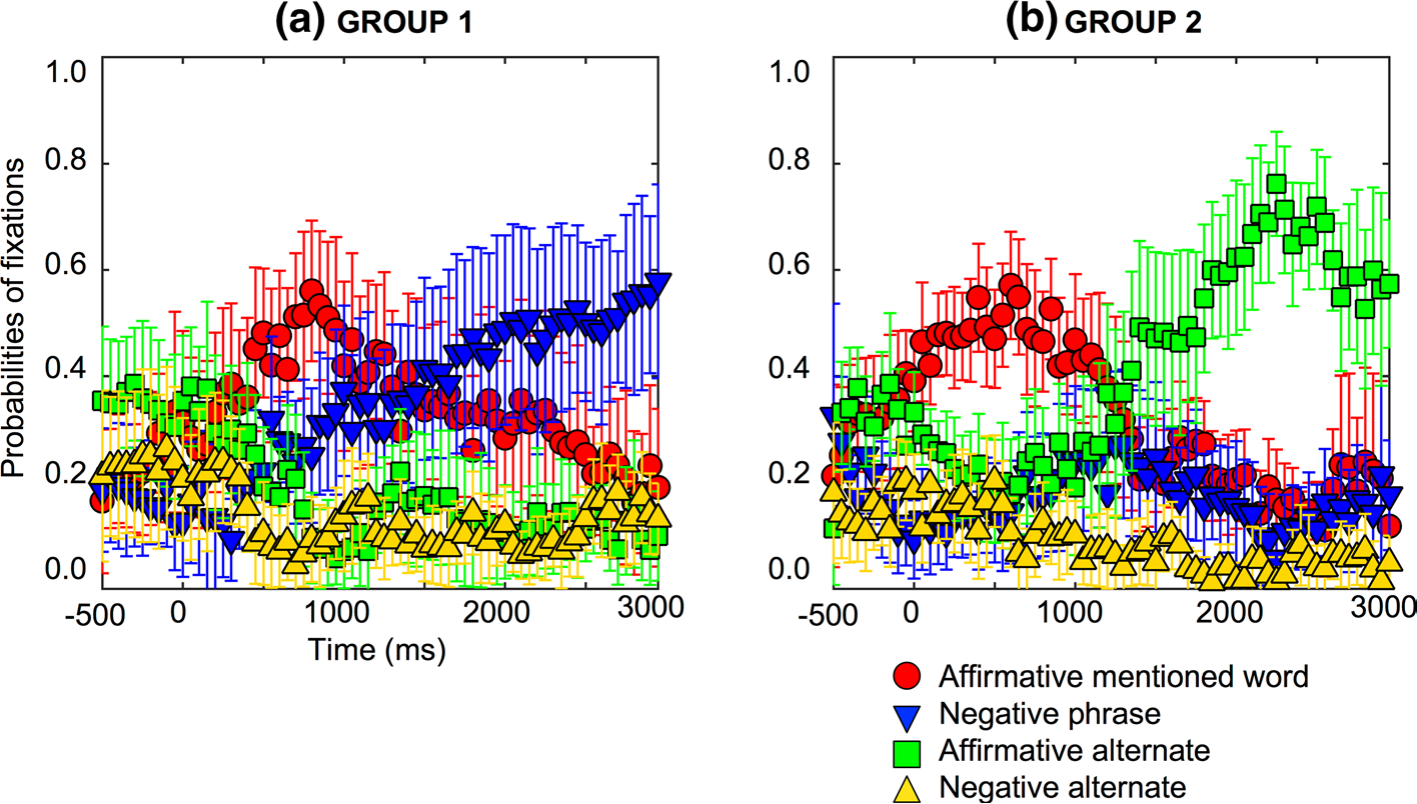
Individual difference probabilities of fixations for affirmative counterfactuals for one subset group of 12 participants who looked at the negative phrase, e.g., “no salad” (a), and a second subset group of 12 participants who looked at the affirmative alternate, e.g., “soup” (b). Error bars are 95% confidence intervals within participants (see Morey, 2008; O’Brien & Cousineau, 2014)

The question that arises here is whether people represent the negative phrase and the affirmative alternate or whether it is a matter of individual differences like the negative causal assertions. This was studied participant by participant and two different processes were found. The participants were split in two groups, those who looked at the explicit negation more than the alternate (n = 12); and those who looked at the alternate more than the explicit negation (n = 12). Six participants were excluded from this analysis because they did not satisfy any of the criteria (see Fig. 3).

*GROUP 1: Pattern of fixations on the negative phrase when understanding the affirmative counterfactuals.* Participants focused on the four words on the screen with probabilities of fixation of 0.1 to 0.4, as Fig. 3a shows. The probabilities of fixation on the negative phrase (e.g., no salad) increased from 1600 ms (*pFDRcorr* = 0.0279), while the affirmative mentioned word (e.g., salad), the affirmative alternative (e.g., soup), and the negative alternate, (e.g., no soup) remained relatively constant, and showed no differences compared to the baseline throughout. In sum, this group of participants just looked at the negative phrase (e.g., no salad) in the affirmative counterfactuals even when the explicit negation was not mentioned, therefore, the representation of the explicit negation is associated to the conceptual meaning.

*GROUP 2: Pattern of fixations on the affirmative alternate when understanding the affirmative counterfactuals.* Participants focused on the four words on the screen with probabilities of fixation of 0.1 to 0.4, as Fig. 3b shows. The probabilities of fixation on the affirmative mentioned word (e.g., salad) started to decrease from 1950 ms (*pFDRcorr* = 0.0161), while the negative phrase (e.g., no salad) remained relatively constant, and showed no differences compared to the baseline throughout. Fixations on the affirmative alternate (e.g., soup) increased from 1900 ms (*pFDRcorr* = 0.0183), and fixations decreased on the negative alternate, (e.g., no soup, from 1050 ms, pFDRcorr = 0.0276). Hence, this group of participants looked at the affirmative alternate (e.g., soup) in the affirmative counterfactuals.

The results showed two ways of processing affirmative counterfactuals quite similar to negative causal assertions. In fact, they can be considered a sort of implicit negation, since the factual situation refers to a fact that does not happen. In this case, people needed to represent the factual situation between 1600 ms (by the negative phrase) and 1900 ms (by the affirmative alternate). Although, an increase in visual attention on the conjecture (the affirmative mentioned word) is observed (see Fig. 3), it was not significant, maybe because of the small sample. Other studies have found that people represent the conjecture and the factual situation to understand the counterfactuals, but the probabilities of fixation on the factual situation are higher than for the conjecture (Orenes et al., 2021). In sum, people represented the factual situation by the negative phrase or the affirmative alternate for affirmative counterfactuals, and both processes were slower than negative counterfactuals. These results corroborated the idea that the inference from not-A to A for negative counterfactuals is faster than from A to not-A for affirmative counterfactuals (Wason, 1965).

### Growth Curve Analysis

Growth curve analyses (Mirman, 2014) were used to compare between conditions and dissociate the integration of negation from the superficial lexical features. This analysis takes the whole period of time through the trajectory of the curve, not point by point, as t-test against baseline. The curve has four elements: the intercept (total fixations), the linear or slope (how fast the curve increases), the quadratic (rise and fall rate of fixation ratios around the central inflection point), and the cubic (the sharpness of the two peaks). The chosen period was the time course of fixations from 400 to 2000 ms after the mentioned word onset, e.g., “salad” (from the earliest increase of word-driven fixations to when target fixation had plateaued). The overall time course of fixations was captured with third-order (cubic) orthogonal polynomial terms and fixed effects of condition within participants on all time terms. The model also included participant and participant-by-condition random effects on all time terms. Statistical significance (p-values) for individual parameter estimates was assessed using the normal approximation (treating the t-value as a z-value). All analyses were carried out in R version 3.5.3 using the lme4 package (for a similar analysis, see Orenes et al., 2014).

**Fig. 4:**
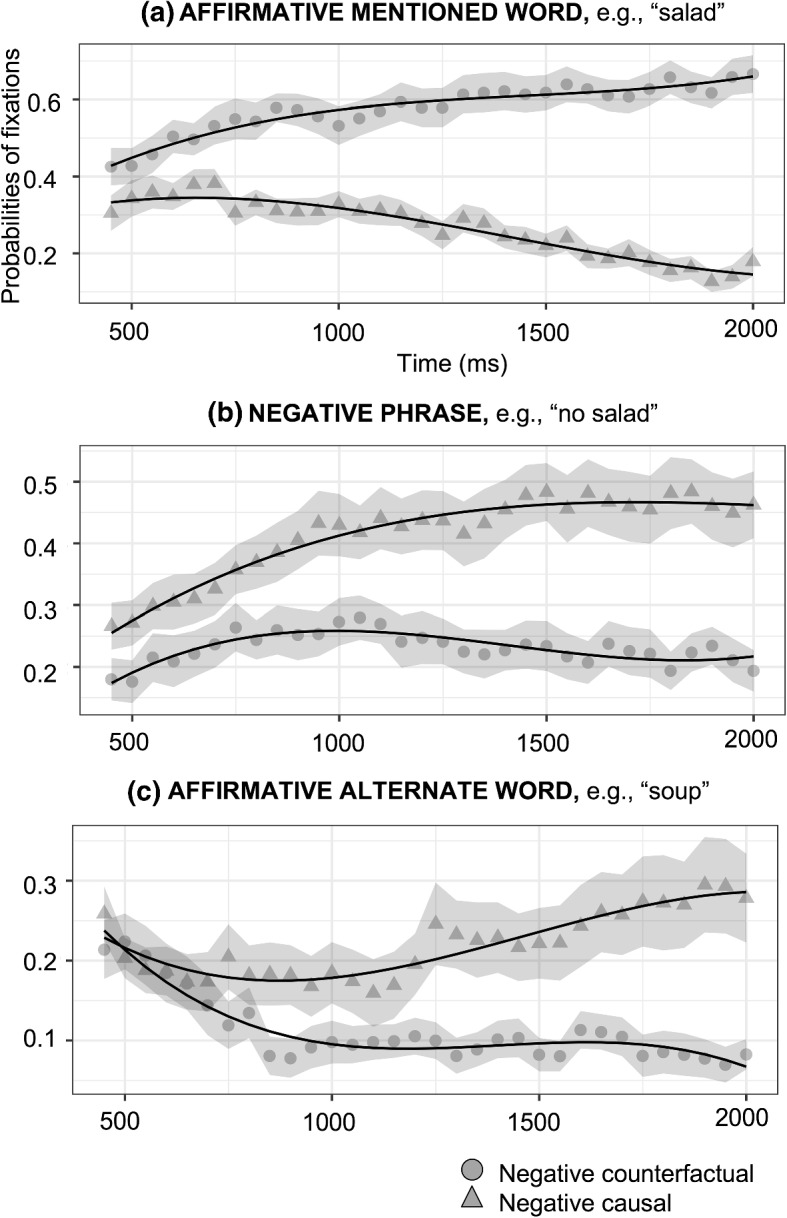
Probabilities of fixations on negative causal assertions (triangles) and negative counterfactuals (circles, shading indicates 95% confidence intervals within participants; see Morey, 2008; O’Brien & Cousineau, 2014) and growth curve model fits (lines) for effect of type of assertions (Condition) on the time course of fixations for the affirmative mentioned word (a), negative phrase (b), and affirmative alternate word (c). Model equation: *lmer(meanFix* ~ *(ot*_*1*_ + *ot*_*2*_ + *ot*_*3*_)*Condition + *(ot*_*1*_ + *ot*_*2*_ + *ot*_*3*_* | Subject)* + *(ot*_*1*_ + *ot*_*2*_ + *ot*_*3*_* | Subject:Condition), control* = *lmerControl (optimizer* = *"bobyqa"), data* = *Datafile, REML* = *FALSE)*. ot_1_ = Linear term of the curve; ot_2_ = Quadratic term; ot_3_ = Cubic term

*Negative causal vs negative counterfactual.* The fixations on the affirmative mentioned word (e.g., salad), the negative phrase (e.g., no salad) and the affirmative alternate (e.g., soup) are compared between the negative causal assertions (e.g., “Because he was *not* hungry, he did *not* order a salad”) and the negative counterfactuals (e.g., “If he had *not* been hungry, he would *not* have ordered a salad”). In both sentences, people hear “no salad,” but they think about the fact it describes, which could either be the explicit negation (e.g., no salad) or the affirmative alternate (e.g., soup) for negative causal assertions and “salad” for negative counterfactuals. For the affirmative mentioned word (e.g., salad), there was an effect on the intercept (*Estimate* = − 0.312, *SE* = 0.039, *p* < 0.001), indicating more looks towards the negative counterfactuals than negative causal assertions; and an effect on the linear term (*Estimate* = − 0.701, *SE* = 0.100, *p* < 0.001), indicating that the slope was steeper for the negative counterfactuals than the negative causal assertions. For the negative phrase (e.g., no salad), there was an effect on the intercept (*Estimate* = 0.183, *SE* = 0.035, *p* < 0.001), indicating higher fixations for the negative causal assertions, while for the negative counterfactuals their looks remained relatively constant for the entire duration; and an effect on the linear term (*Estimate* = 0.340, *SE* = 0.111, *p* = 0.002), indicating that the slope was steeper for the negative causal assertions than the negative counterfactuals. For the alternate word (e.g., soup), there was an effect on the intercept (*Estimate* = 0.107, *SE* = 0.031, *p* < 0.001), indicating a higher fixation probability for the negative causal assertions than the negative counterfactuals; and an effect on the linear (*Estimate* = 0.348, *SE* = 0.111, *p* = 0.001), indicating that the slope was steeper for the negative causal assertions than the negative counterfactuals. These results show that people mostly looked at the factual situation, the affirmative mentioned word (e.g., salad) for negative counterfactuals and either the negative phrase (e.g., no salad) or the affirmative alternate (e.g., soup) for negative causal assertions (see Fig. 4).

**Fig. 5:**
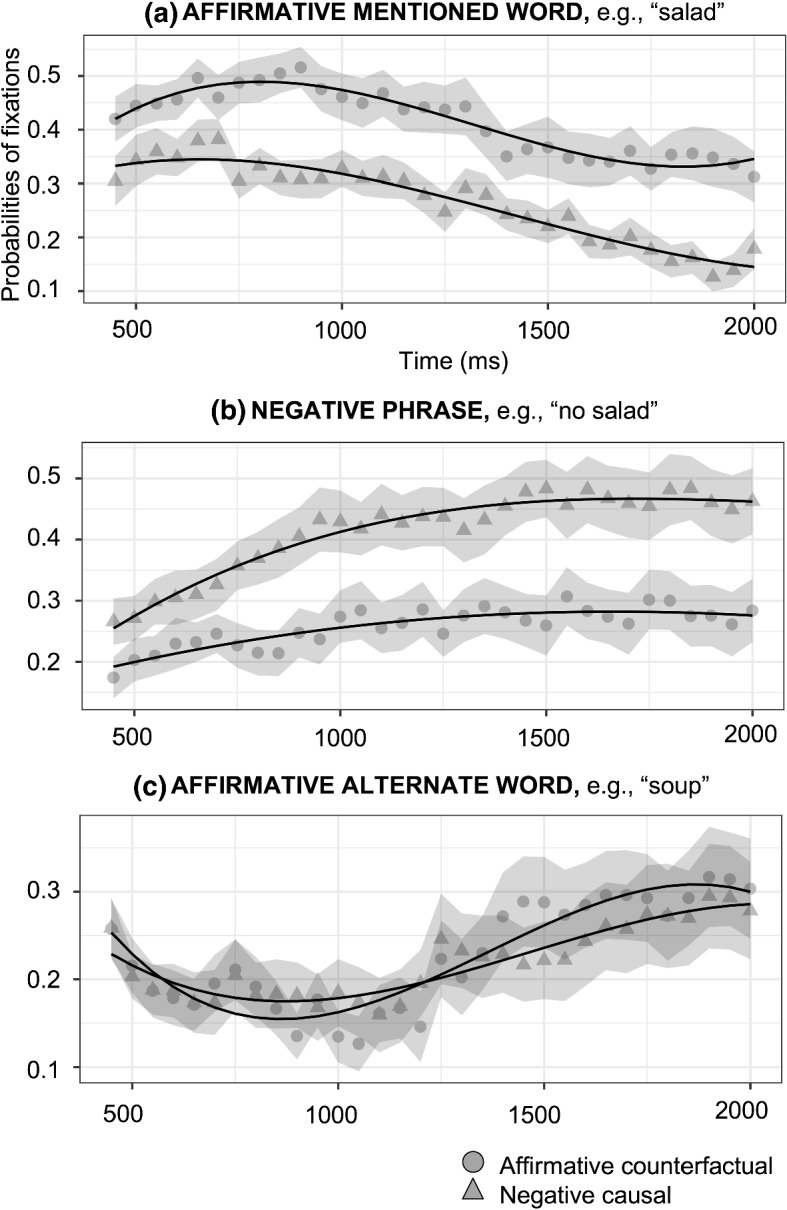
Probabilities of fixations on negative causal assertions (triangles) and affirmative counterfactuals (circles, shading indicates 95% confidence intervals within participants; see Morey, 2008; O’Brien & Cousineau, 2014) and growth curve model fits (lines) for effect of type of assertions (Condition) on the time course of fixations for the affirmative mentioned word (a), negative phrase (b), and affirmative alternate word (c)

*Negative causal vs affirmative counterfactual.* It is compared the fixations on the affirmative mentioned word (e.g., salad), the negative phrase (e.g., no salad) and the affirmative alternate (e.g., soup) between the negative causal assertions and the affirmative counterfactuals to test the effects related to the meanings encoded by these sentences. In this case, people heard different words, “no salad” for negative causal assertions (e.g., “Because he was *not* hungry, he did *not* order a salad”) and “salad” for affirmative counterfactuals (e.g., “If he had been hungry, he would have ordered a salad”), but the facts are the same for both types of sentences, either the explicit negation (e.g., no salad) or the affirmative alternate (e.g., soup). For the affirmative mentioned word (e.g., salad), there was only an effect on the intercept (*Estimate* = − 0.149, *SE* = 0.030, *p* < 0.001), indicating higher fixation probability for the affirmative counterfactuals than negative causal assertions. This could indicate that people look more at the word corresponding to the conjecture for affirmative counterfactuals than for the negated situation for negative causal assertions. For the negative phrase (e.g., no salad), there was an effect on the intercept (*Estimate* = 0.156, *SE* = 0.026, *p* < 0.001), indicating higher fixation probability for the negative causal assertions than the affirmative counterfactuals. This could be due to people looking at the negative phrase (e.g., no salad) much earlier for the negative causal assertions than for the affirmative counterfactuals, therefore the total fixations is greater. There were no differences between both types of sentences for the affirmative alternate word (e.g., soup). It could indicate that the number of fixations is similar in both sentences and overlap in time, which supports that the representation of the alternate is late (see Fig. 5).

## General Discussion

The results of the present study corroborated that there are at least two different ways to process negative sentences that are related to its representation: the representation of the explicit negation (the negative phrase) is fast, while its alternate (the affirmative alternate) is slow because an inference is necessary. The advantage of the representation of the explicit negation could accelerate its processing and save cognitive resources, but this symbolic representation is harder to remember and understand (Fillenbaum, 1966; Hasson & Glucksberg 2006; Kaup, 2001; Kaup & Zwaan, 2003; Lea & Mulligan, 2002; MacDonald & Just, 1989; Mayo et al., 2014). This leads people to represent the alternate when it is available, as although its processing is slow, its comprehension and memory improve (Beltrán et al., 2008; Orenes et al., 2014). These results fit well with the model theory because it predicts at least two representations for negation depending on the availability of the alternate: iconic that corresponds to the alternate and symbolic, that corresponds to the explicit negation (e.g., Beltrán et al., 2008; Khemlani et al., 2012; 2014; Orenes et al., 2014). At the same time, it poses problems to the embodied cognition account that assumes that negation cannot be represented explicitly and that the only way to represent negation is by the alternate (see Kaup et al., 2006; Kaup & Zwaan, 2003). However, the results of the present study show that both, the alternate is not necessary to understand negation and that it can maintain its own meaning in symbolic format. These results cannot be explained by the pragmatic approach which predicts individuals always represent the actual situation immediately, as eye gaze showed late representations of the alternate. In this experiment, no context (either binary or multiple) was presented to modulate negation processing. Therefore, these two ways to process negation are mostly predicted by the model theory (see similar approaches in Meteyard et al., 2012).

The present results show that when people look at the alternate of the negation, they do not increase first their looks on the explicit negation. Indeed, both types of processing seem to be opposite, that is, when people look at the explicit negation, they do not look at the alternate, and vice versa. Previous studies showed the same results (Orenes et al., 2014; Orenes et al., 2021) and they were interpreted by the previous context (i.e., binary: “he did not know whether to eat salad or soup”) that made people look at only those two words (salad and soup) before the target sentence and ignore the negative phrases. However, the results of the present study remain identical without any previous context to highlight any particular word. It is surprising because people infer the alternate “soup” from the negative sentence “he did *not* order salad,” therefore it would be expected that people would look at “not salad” first and next “soup,” but this inference or operation is not caught by the eye-tracker. People look directly at the actual situation. This is corroborated by negative counterfactuals (e.g., “If he had *not* been hungry, he would *not* have ordered a salad”) in which people look at the actual situation (e.g., salad) very early on without increasing their looks toward the negative phrase (e.g., no salad). In sum, eye tracking data show that the looks are guided by sentence meaning rather than by superficial lexical features.

There is no evidence of the double processing of negation in the negative causal assertions. People did not look at the negated situation, e.g., “salad,” that is often thought of as the first step of negation processing. This finding is consistent with the incremental comprehension of negation that is accepted by a pragmatic approach, in which the initial comprehension of negative sentences matches their “final” interpretation (as reflected in offline judgments; Nieuwland, 2016; Nieuwland & Kuperberg, 2008). It cannot be discarded that this could be a limitation of the eyes that cannot detect it. However, fixations detected the double processing of counterfactuals (e.g., “If he had been hungry, he would have ordered a salad”). People looked at the conjecture (e.g., salad) and the factual situation when understanding counterfactuals. Therefore, it seems that the double processing is essential to represent the counterfactual meaning (see Byrne, 2005; 2016). Regarding the negative causal assertions, it is also true that the representation of the explicit negation (e.g., no salad) is really fast, around 400–600 ms, and the representation of the negated situation should be earlier, then it is possible that the eyes cannot catch the double processing of negation since 180–200 ms are necessary for saccade programming (Martin et al., 1993). Future research should focus on this issue.

In sum, there are at least two ways of processing negation and it seems that the negation operator is integrated immediately, while the representation of its alternate takes extra time. Both processes could explain the conflicting observations in previous comprehension studies about the incremental processing of negation from the pragmatic accounts or the idea that the integration of negation is delayed from the double processing account.
